# Muscular Power during a Lifting Task Increases after Three Months of Resistance Training in Overweight and Obese Individuals

**DOI:** 10.3390/sports5020035

**Published:** 2017-06-08

**Authors:** Erika Zemková, Ol’ga Kyselovičová, Michal Jeleň, Zuzana Kováčiková, Gábor Ollé, Gabriela Štefániková, Tomáš Vilman, Miroslav Baláž, Timea Kurdiová, Jozef Ukropec, Barbara Ukropcová

**Affiliations:** 1 Faculty of Physical Education and Sports, Comenius University in Bratislava, Bratislava 81469, Slovakia; kyselovicova@fsport.uniba.sk (O.K.); michaljelen666@yahoo.com (M.J.); kovacikzuz@gmail.com (Z.K.); gabor_olle@yahoo.com (G.O.); gabi.stefanikova@gmail.com (G.Š.); tomasvilman@gmail.com (T.V.); 2 Institute of Experimental Endocrinology, Slovak Academy of Sciences, Bratislava 83306, Slovakia; Miroslav.Balaz@savba.sk (M.B.); Timea.Kurdiova@savba.sk (T.K.); Jozef.Ukropec@savba.sk (J.U.); Barbara.Ukropcova@savba.sk (B.U.); 3 Institute of Pathological Physiology, Faculty of Medicine, Comenius University in Bratislava, Bratislava 81372, Slovakia

**Keywords:** deadlift, deadlift high pull, muscle power, obesity, testing and training

## Abstract

Background: This study evaluates the effect on power produced during a modified lifting task in the overweight and obese after three months of either resistance or aerobic training. Methods: Seventeen male subjects divided randomly into two groups performed deadlift and deadlift high pull, both with increasing weights up to maximal power, prior to and after the training programs (three sessions per week). Results: Their mean power increased significantly during the deadlift at 20 kg (14.3%, *p* = 0.026), 30 kg (17.7%, *p* = 0.008), 40 kg (16.5%, *p* = 0.011), 50 kg (14.5%, *p* = 0.020), and 60 kg (14.3%, *p* = 0.021) and during the deadlift high pull at 30 kg (9.9%, *p* = 0.037), 40 kg (10.1%, *p* = 0.035), and 50 kg (8.2%, *p* = 0.044) after the resistance training. However, the group that participated in the aerobic training failed to show any significant changes in power performance during either the deadlift or deadlift high pull. Conclusion: Three months of resistance training enhances power outputs during a lifting task with weights from 30 to 50 kg (~40–60% of 1-repetition maximum) in the overweight and obese. Because this test was sensitive in revealing pre-post training changes in lifting performance, it should be implemented in the functional diagnostics for overweight and obese individuals and also complement existing testing methods.

## 1. Introduction

Obesity is often identified as a factor associated with functional task limitations [1,2,3,4]. The reduced physical capacities range from attempting normal activities such as rising from a chair, lifting, walking and carrying bags to assorted other occupational tasks. The obese are often less efficient in manual work-related tasks that demand prolonged physical effort and/or require dynamic transfer of torque and momentum throughout the kinetic chain.

This is especially relevant during lifting tasks. Obesity itself represents a persistent cause of overload on the spine and related nervous, discal and ligamentous structures [5]. The spine has limited flexibility and increased dorsal stiffness [6,7], which affects the execution of job tasks involving the trunk.

The functioning of the central core of the body is important for stabilization and force generation during a lifting task. The ‘core’ has been described as a box with the abdominals in the front, paraspinals and gluteals in the back, the diaphragm as the roof, and the pelvic floor and hip girdle musculature as the bottom [8]. While the term ‘core strength’ refers to the strength of these muscles, ‘core stability’ is the ability to control the position and motion of the trunk over the pelvis and leg to allow optimum production, transfer and control of force and motion to the terminal segment in integrated kinetic chain activities [9]. Despite the widespread promotion of core strengthening and stabilization exercises, there is a lack of information regarding the efficiency of resistance training for the improvement of physical fitness and the prevention or rehabilitation of musculoskeletal disorders.

Commonly, resistance exercises using stacked weight machines provide the greatest stability and therefore are utilized for beginners who are unfamiliar with free weight exercise techniques. Such fixed-form training allows maximal force to be exerted by the targeted muscle group. However, muscles rarely function in such an isolated manner during a lifting task. While a person performs a movement (e.g., squat, deadlift), the local stabilizers help to maintain mechanical ability and posture of the lumbar spine and the global muscles function to balance the external load that is being applied to the trunk region and generate force in order to maintain the range of motion for the exercise [10]. Hamlyn et al. [11] demonstrated that high trunk muscle activation is needed to stabilize external resistance during traditional weight-training exercises such as the squat and deadlift. Their results showed that the use of moderately high (80% 1-RM) intensity resistance while performing the squat and deadlift could provide greater dorsal trunk activation than similar exercises without external resistance. These findings indicate that ground-based free-weight lifts are more specific to many activities of daily living and occupational tasks. Similar to these activities, the deadlift necessitates the integration of vertebral muscles for mobilization and stabilization. In order to both dynamically lift the weight off the floor and stabilize the thoracic and lumbar vertebrae, the upper back muscles are recruited, resulting in a greater activation of the upper lumbar erector spinae [11]. Therefore, lifting exercises should be the foundation of functional training and testing not only for athletes, but also for those whose work entails lifting tasks.

However, both training and testing methods for the core musculature need to be structured differently, based on the health status and training goals of the athlete and nonathlete [12]. This is especially relevant for the obese, where abnormal mechanics on body movements due to excess weight could account for the high incidence of musculoskeletal disorders when performing manual handling. Traditionally, maximal isometric strength has been evaluated despite manual material-handling tasks requiring a production of power in addition to a coordinated multilink movement. In such a case, testing muscle power would represent a more appropriate alternative. The task of the subjects is to perform resistance exercises with weights increasing stepwise up to a maximal power. These maximal values of power during typical resistance exercises, such as bench presses or squats, occur at intermediate velocities when lifting moderate weights, i.e., 50–60% 1-RM [13]. During the deadlift high pull with free weights or on the Smith machine, maximal values of power are at about 80% and 70% 1-RM in healthy young adults [14]. This approach can be used for the evaluation of the effects of training in the overweight and obese. Indeed, resistance training was found to be efficient in increasing upper and lower body muscle power, whereas there were no significant changes in peak force and the rate of force development measured during maximum voluntary isometric contraction [15].

Though it is generally accepted that improvement in muscle power may be more efficient when resistance exercises are included in the training program, this is often neglected in practice. This is especially the case for some groups including the obese, for whom aerobic exercises are usually recommended. In addition, there is a scarcity of literature on changes in power performance during exercises that best simulate lifting (e.g., a deadlift high pull) following resistance training in the overweight and obese. We assumed that these individuals would be able to enhance their power output not only during the deadlift, but also during the deadlift high pull, which involves working the major muscle groups in a more demanding and coordinated manner. This study evaluates the effect of three months of resistance and aerobic training programs on power produced during a lifting task in the form of a deadlift high pull in overweight and obese individuals.

## 2. Material and Methods

### 2.1. Participants

Seventeen male overweight and obese subjects (age 37.9 ± 5.1 years, height 182.6 ± 5.8 cm, weight 104.4 ± 14.8 kg) volunteered to participate in the study. All the participants were recreationally active, but were not involved in any form of aerobic, strength and/or power training. Habitual free-living ambulatory activity was defined as daily life activities requiring more energy than three times the resting metabolic rate (>3METs). Participants were asked to keep their regular eating habits during the study. Body weight and height were used to calculate BMI (kg·m^−2^). Bioelectric impedance was used to evaluate total and visceral adiposity and to estimate lean body mass (Omron BF511, Omron Healthcare LTD., Matsusaka, Japan). Resting energy expenditure was measured after an overnight fast and following 30 min bed rest by open-circuit indirect calorimetry using the Ergostik system (Geratherm Respiratory, Bad Kissingen, Germany) for a period of 30 min. Volume and dynamics/intensity of daily ambulatory activity were determined with accelerometers (Lifecorder PLUS, Kenz, Nagoya, Japan) continuously within the three months of the intervention.

Participants were all fully informed regarding the procedures and the possible risks, and provided witnessed written informed consent prior to the study. The study was approved by the Ethics Committee of the University Hospital Bratislava at Comenius University in Bratislava and with the Ethics Committee of the Bratislava Region Office, and is conforming to the ethical guidelines of the 2000 Helsinki declaration [16].

### 2.2. Testing and Training

The participants were randomly divided into two groups. While the first group underwent resistance training, the second group performed aerobic training for a period of three months (three sessions/week). Exercise professionals supervised all training sessions. Adherence to the training program was monitored and regularly encouraged.

Prior to and after completing the training, participants were required to perform a deadlift and a deadlift high pull with increasing weights in a random order. After a standardized warm-up (i.e. dynamic flexibility and stretching routine), they were exposed to familiarization trials (i.e., 2–3 sub-maximal effort trials of both exercises with a barbell weight). Following this, they performed two repetitions of the deadlift and the deadlift high pull with maximal effort in the lifting phase. The weight increased stepwise up to a maximal power, as previously described [14]. The more enhanced of the two attempts were selected for analysis. The FiTRO Dyne Premium (FiTRONiC, Bratislava, Slovakia) monitored basic biomechanical parameters. As shown previously, the intraclass correlation coefficients for peak power and mean power during deadlift high pull above 0.80, along with no significant differences between the test results obtained on the first and second test sessions, signify good reliability [14]. However, SEM > 10% for peak power and SEM < 10% for mean power during deadlift high pull with free weights as well as on the Smith machine indicate that the latter represents a more reliable parameter [14]. Therefore mean values of power were used for data analysis in the present study.

Aerobic performance and cardiovascular health status were assessed by a cardiologist in the recruitment process, and after completing the three-month intervention. Each session began with a standardized warm-up (i.e., dynamic flexibility and stretching routine) in duration of 10–15 min and ended with some cool down stretching exercises of about 5 min. The total duration of each training session was one hour. Exercise professionals designed and conducted the training program. During the workout, aerobic dancing (a complex of exercises combining aerobics with dance steps), running and/or jogging, and spinning (a low-impact exercise in terms of lower pressure on joints as compared to former aerobic or running exercises, because it employs a stationary bike) were alternated three times per week (Monday, Wednesday and Friday) for three months. During each session the exercise intensity was monitored using a Polar RS300X (Polar, Finland). Intensity was maintained at 70–85% of maximal heart rate.

The resistance-training program was designed according to the individual strength assessments at the beginning of the study. Subjects performed the periodized strength-training program three times per week (Monday, Wednesday and Friday) for 12 consecutive weeks via the use of equipment, free weight and machine resistance exercises. Participants were informed about training methods and the training equipment to ensure the safety of the workouts. The aim of the resistance-training program was to enhance maximal strength as well as explosive power when possible. Each session consisted of a brief warm-up, followed by exercises for strengthening the major muscle groups. Within 2–3 workouts, the optimal weight was selected. This period gave the participants the opportunity to practice technique and experiment with different resistances. The strength-training program was designed according to the fundamental principles that ensure a progressive overload of the musculature. Progress was made through increasing the amount of weight, increasing the number of repetitions and sets, decreasing the amount of rest time between sets, or a combination of any of these according to the subject’s physical fitness. The exercises utilized standard free weights and equipment that is available in most gyms. This approach allowed participants to become familiar with the beginners weight-training workout routine that can be easily followed after completion of the study.

Structure of the sessions: Each session began with a general warm-up of 10–15 min and ended with static stretching of approximately 5 min. The total duration of training session was 60–70 min. Four to five specific resistance exercises were applied twice per week (Monday and Wednesday). The sequence of exercises was maintained throughout the study. While the Monday served as an “upper body” workout focusing on chest, shoulder and arm muscles, the Wednesday routine exercised the “lower body”: thigh, hips and back muscles. The examples of strength training sessions 1 and 2 are outlined in Table 1. The exercises of the third session (Friday) activated a larger muscle mass (multijoint) and core muscles (push-ups, squats, sit-ups, swing, seated lat pull-down, shoulder press, prone plank). The third session was carried out as the functional training via circuits using open chain and closed chain exercises (Table 2).

Training program: All training sessions were carried out individually and trainees were under constant supervision. An experienced expert was present during every training session to ensure the exercises were performed correctly. The weight loads were set differently and mainly reflected the individuals’ level of strength, performance, and acute or chronic health problems. In each session, the subjects completed a specific number of repetitions and sets depending on the intensity for that workout session (Table 3). The training progression incorporated three levels of difficulty by increasing the weight load (percent 1-RM), repetitions.set^−1^, sets.exercise^−1^ and rest intervals. Individual assessments prior to the beginning of the program determined exercise selection and load gradation-intensity. The initial four weeks (phase 1) were used as an anatomical adaptation and familiarization phase, with moderate intensity and volume. In the second phase (weeks 5–8), the program used a gradual linear increase in intensity and duration. After eight weeks, the subjects who completed the exercises correctly, progressed to a third difficulty level (weeks 9–12).

### 2.3. Statistical Analyses

Data analyses were performed using statistical program SPSS for Windows version 18.0 (SPSS, Inc., Chicago, IL, USA) and a two-way analysis of variance (ANOVA) with repeated measures. Factors included time (pre-training and post-training) × group (resistance and aerobic). When significant changes were revealed (*p* ≤ 0.05), the differences between mean values of power before and after training were compared by a one-tailed Student’s *t*-test, which was corrected by Bonferroni adjustments. The level of significance was set at 0.05. Effect sizes (ES) were determined by calculating Cohen’s d values [17]. The values 0–0.19 were considered trivial, while 0.20–0.49 were small, 0.50–0.79 were medium, and 0.80 and greater were large. Group data were presented as means ± standard deviations (SDs).

## 3. Results

There were no significant pre-post intervention changes in anthropometric and body composition variables (body mass from 104.4 ± 14.8 kg to 104.4 ± 15.4 kg, BMI from 31.2 ± 3.7 kg·m^−2^ to 31.3 ± 4.0 kg·m^−2^, and body fat from 30.6% ± 4.7% to 29.3% ± 5.1%).

Mean power during both the deadlift and the deadlift high pull increased from lower weights up to the maximum. During deadlift one participant achieved maximal power at 60 kg (340.2 W), five at 80 kg (531.8 ± 43.4 W), four at 85 kg (555.2 ± 54.8 W), three at 90 kg (527.6 ± 42.5 W), one at 95 kg (493.0 W), and three at 100 kg (517.0 ± 76.8 W). During the deadlift high pull three participants achieved maximal values of power at 50 kg (393.1 ± 58.1 W), six at 60 kg (577.1 ± 54.7 W), six at 65 kg (576.4 ± 53.9 W), and two at 70 kg (649.1 ± 30.4 W).

**Figure 1:**
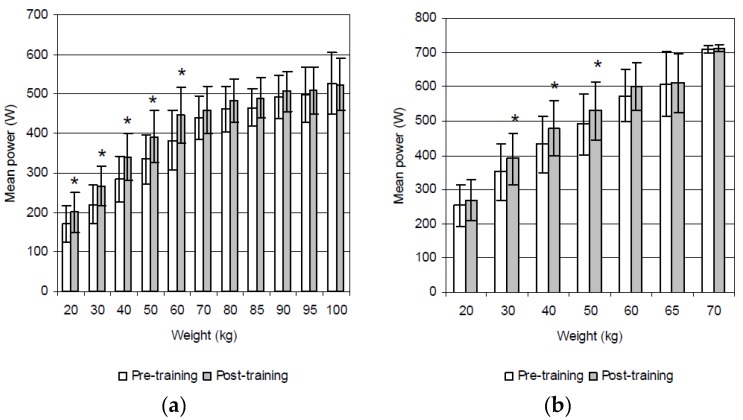
Mean power during the (**a**) deadlift and (**b**) deadlift high pull prior to and after resistance training (* *p* < 0.05).

**Figure 2:**
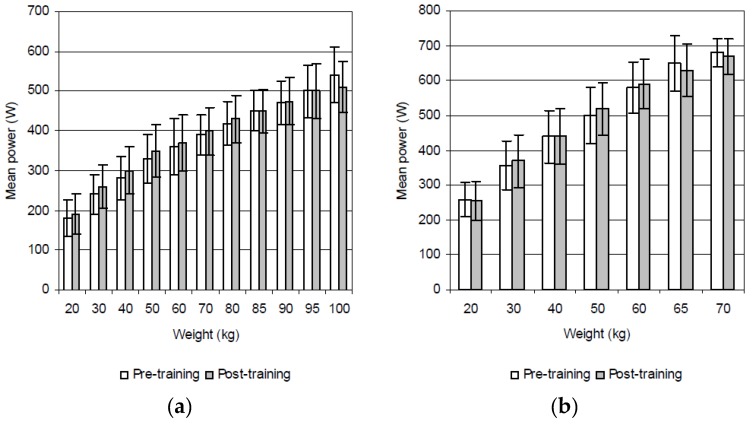
Mean power during the (**a**) deadlift and (**b**) deadlift high pull prior to and after aerobic training.

The ANOVA revealed a significant main effect for group × time (*p* < 0.01), i.e., a significant increase in power output following the resistance, but not after the aerobic training. Following the resistance training, the mean power during the deadlift increased significantly at 20 kg (14.3%, *p* = 0.026; ES = 0.7), 30 kg (17.7%, *p* = 0.008; ES = 1.0), 40 kg (16.5%, *p* = 0.011; ES = 0.9), 50 kg (14.5%, *p* = 0.020; ES = 0.9), and 60 kg (14.3%, *p* = 0.021; ES = 0.8) (Figure 1a). These values also increased significantly during the deadlift high pull at 30 kg (9.9%, *p* = 0.037; ES = 0.5), 40 kg (10.1%, *p* = 0.035; ES = 0.6), and 50 kg (8.2%, *p* = 0.044; ES = 0.5) (Figure 1b). As expected, the group that participated in the aerobic training failed to display any significant improvement in power performance during either the deadlift and/or the deadlift high pull (Figure 2a,b).

## 4. Discussion

Previous study revealed a significant association between the power produced during deadlift and deadlift high pull (r = 0.627 for peak values and 0.639 for mean values, *p* < 0.05) [14]. Following the resistance training, mean power during the deadlift increased significantly at 20 kg (14.3%), 30 kg (17.7%), 40 kg (16.5%), 50 kg (14.5%), and 60 kg (14.3%). These values increased significantly also during the deadlift high pull at 30 kg (9.9%), 40 kg (10.1%), and 50 kg (8.2%). Enhancement of power outputs during these exercises may be attributed to the applied training consisting of ground-based free-weight exercises in addition to exercises on strength-training machines. The activation of the muscles that function as stabilizers was greater during free-weight than fixed-form training [18]. Performing exercises such as ground-based free-weight lifts that require muscular stabilization of posture [19] might have contributed to improving power performance mainly during the deadlift high pull. This exercise imposes greater demands on neuromuscular coordination than the deadlift alone, especially for the overweight and obese. Given the low physical fitness in these subjects, who all had a predominantly sedentary lifestyle at the beginning of the study, it is likely that the acquisition of a certain degree of motor skills contributed toward the enhancement of muscle power during a modified lifting task in a form of the deadlift high pull. On the other hand, the aerobic group failed to show any significant improvements because the training stimuli were not sufficient to enhance muscle power.

Functional task limitations and changes in the mechanical strategies used to complete functional tasks may be related independently to increased mass and/or dimensions [20]. Increased mass is not always detrimental. In lifting, the increased mass appears to be used positively as part of the strategy of the lifting technique. For the obese, holding a box in a static posture results in a higher perceived exertion [21], but obesity does not affect the maximum acceptable weights of the lift [22]. In the present study, some of the overweight and obese participants were able to achieve maximal power at 100 kg during the deadlift and at 70 kg during the deadlift high pull.

Obese persons may benefit from a resistance training program, which improves muscular strength and favorably affects functional tasks [23]. Recently, Villareal et al. [24] reported that weight loss plus combined aerobic and resistance exercise is most effective for improving the functional status of obese older adults. However, there are only a few published studies evaluating the effects of resistance and aerobic training on lifting performance in overweight and obese individuals in comparison with those demonstrating improvements of their upper and lower body muscle strength and power. For instance, Kraemer et al. [25] discovered that maximum strength, as determined by 1-RM testing in the bench press and squat exercise, significantly increased for a diet group of overweight men that performed both aerobic and strength training three times per week for 12 weeks in both the bench press (+19.6%) and squat exercise (+32.6%). Similarly, obese women following 12-week resistance training significantly improved in 1-RM tests of hip abductors, quadriceps, biceps, and pectorals compared with those in the control group [26]. Hip abductor muscle strength was the only area where there was a significant increase in resistance compared with the aerobic exercise group. However, a study by Sartorio et al. [27] identified a significant increase in the absolute lower limb anaerobic power output evaluated by means of a jumping test after three weeks of aerobic (A: 13.7%) and aerobic plus strength training (AS: 18.1%), without any substantial difference between A and AS. The addition of strength training to A conditioning increased maximum strength. The maximum strength increase after the body weight reduction program determined by one maximal repetition test of lower and upper limb muscle groups was significantly greater in the AS group, ranging from 11.4% to 25.4% (A) and from 26.7 to 41.8% (AS).

Because energy-restricted diet is usually a part of interventions in obese and overweight people, concerns exist about the effect of weight loss on muscle function. Willis et al. [28] demonstrated that aerobic training is the optimal mode of exercise for reducing both fat and body mass, while a program including resistance training is necessary for increasing lean mass in the middle-aged and the overweight/obese. Kim et al. [29] identified that the weight-loss program led to a 14.1% weight loss accompanied by significant loss of leg muscle mass, static maximal muscle strength, dynamic maximal muscle strength and dynamic muscle endurance, but not with significant loss of dynamic muscle power. Decline of muscle strength was related to a decrease in muscle mass, but not completely dependent on a decrease in muscle mass. Body weight-normalized muscle strength increased significantly.

A weight-loss dietary regimen in conjunction with aerobic and resistance exercise prevented the normal decline in fat-free mass and muscular power and augmented body composition, maximal strength, and maximum oxygen consumption compared with weight loss induced by diet alone [24]. In study by March et al. [30] a hypocaloric weight loss intervention in older overweight and obese adults led to significant declines in lean body mass and appendicular lean body mass. However, in those assigned to resistance training, leg power significantly improved following the intervention, whereas muscle strength and power was not adversely affected in the other groups (no resistance training, pioglitazone or placebo). Furthermore, older adults with sarcopenic obesity who engaged in the resistance training, aerobic training, and combination training interventions demonstrated increased muscle mass and reduced total fat mass and visceral fat area compared with those without training [31]. The muscle strength performance and serum IGF-1 level in trained groups, especially in the resistance training group, were superior to the control group. According to Nicklas et al. [32], the addition of caloric restriction during resistance training improves mobility and does not compromise other functional adaptations to resistance training. The authors showed that resistance training improves body composition, muscle strength and physical function in the obese. Sartorio et al. [33] reported that a short-term (three-week) mass reduction program (five days/week) entailing exercise (aerobic and strength training) and an energy-restricted diet for the morbid obesity induced significant effects on body weight changes and composition as well as on maximum voluntary total isotonic strength and maximum leg power output per unit body weight. In spite of the positive correlation of maximum voluntary total isotonic strength and maximum leg power output with fat-free mass, there was no detectable relation between the changes in body composition and those in motor performance. The improvement in maximum voluntary total isotonic strength and maximum leg power output was attributable to factors other than muscle mass change. These findings, along with the observations in the present study, support the incorporation of resistance training into obesity treatments regardless of whether caloric restriction is part of the intervention.

Recent guidelines on exercise for weight loss and weight maintenance include resistance training as part of the exercise prescription. Weight lifting or progressive resistance training (PRT) is recommended for the obese by the American College of Sports Medicine [34]. PRT is defined as exercise whereby the resistance against which a muscle generates force is progressively increased over time [35]. It increases muscular size and strength, alters body composition by increasing lean body mass and decreasing visceral and total body fat [36], and leads to changes in neuroendocrine and cardiovascular functions [37]. Resistance training at intensities of between 60% and 100% of the one repetition maximum (1-RM) elicit structural, functional, and metabolic changes in skeletal muscle, with higher intensities leading to greater adaptations [38]. This is beneficial for overweight and obese people who are significantly weaker than normal-weight subjects, despite greater muscle mass [39]. When normalized to body weight, strength is 6–10% lower in those who suffer from obesity as compared with their leaner counterparts. Reduced muscle strength could possibly stem from diminished muscle function, abnormal metabolism (lower oxidative capacity of muscle fibers, despite their hypertrophy), and lower physical activity levels, also exhibited by reduced motor unit activation during exercise [40]. Besides this, the excess weight of obese people has a significant added effect on the musculoskeletal structures of the back, which expose them to a higher risk of developing musculoskeletal disorders during load handling [41]. Resistance training, including exercises strengthening core musculature, is therefore of special importance for this segment of the population.

If possible, muscle power should be measured through a performance test [42]. Measurement of power during exercises such as the deadlift or deadlift high pull using portable diagnostic systems allows for the assessment of lifting performance under non-laboratory conditions. It is a suitable method for both the physically active and for those who lead a more sedentary lifestyle. This study highlights that it may also provide useful data for the evaluation of the efficiency of exercise programs in the overweight and obese.

## 5. Conclusions

Three months of resistance training enhanced power performance during a modified lifting task in overweight and obese individuals. Mean power during deadlift increased significantly at 20 kg (14.3%), 30 kg (17.7%), 40 kg (16.5%), 50 kg (14.5%), and 60 kg (14.3%) after resistance training. The values increased significantly also during the deadlift high pull at 30 kg (9.9%), 40 kg (10.1%), and 50 kg (8.2%). However, the group that participated in the aerobic training failed to show any significant improvement of power performance during either the deadlift and/or the deadlift high pull. Such improvements in lifting performance after resistance training in the overweight and obese may increase quality of life, decrease the risk of falling and reduce health-related consequences.

This is the first study to demonstrate that the deadlift high pull with free weights may be a suitable test for evaluating lifting performance in the overweight and obese. The test was sensitive to changes in power outputs during a modified lifting task following the training.

## Figures and Tables

**Table 1:** An example of training sessions.

**EXERCISE—Monday (Upper Body)**	**LOAD (Repetitions × Sets × Rest)**
CHEST—m. pectoralis	–
Bench Press	8 Reps × 5 Sets × 90 s
Incline Bench Press	8 Reps × 4 Sets × 90 s
ARM—m. triceps brachii	–
Triceps Push Down	10 Reps × 4 Sets × 90 s
ARM—m. deltoid	–
Seated Shoulder Dumbbells Press	10 Rep × 4 Sets × 90 s
**EXERCISE—Wednesday (Lower Body & Back)**	**LOAD (Repetitions × Sets × Rest)**
BACK—m. dorsi	–
Seated Lat Pulldown	12 Reps × 5 Sets × 90 s
Seated Row	10 Reps × 4 Sets × 90 s
HIP/THIGH	–
Seated Leg (knee) Extension	8 Reps × 2 Sets × 90 s
Back Squat	12 Reps × 5 Sets × 90 s
Forward Lunge	10 Reps × 5 Sets × 90 s

**Table 2:** Circuit training sessions—Fridays (functional training).

Week	Load	Rest between Exercises	Circuits	Rest between Circuits
1–4	30 s	30 s	6	120 s
5–8	45 s	30 s	7	90 s
9–12	60 s	No rest	8	60 s

**Table 3:** Average training values for the periodized strength-training program.

Training Program		d·Week^−1^	Intensity (% 1-RM)	Duration (min·Week^−1^)
Phase 1	Week 1	3	57	120
	Week 2	3	57	120
	Week 3	3	62	135
	Week 4	3	62	135
Phase 2	Week 5	3	70	144
	Week 6	3	70	144
	Week 7	3	74	150
	Week 8	3	74	150
Phase 3	Week 9	3	79	168
	Week 10	3	79	168
	Week 11	3	82	180
	Week 12	3	82	180

d·week^−1^ = the number of training days per week; intensity = the average intensity assessed in percent of 1 repetition maximum (RM) for each week; duration = the approximate total workout time of each resistance training sessions.
